# Crystal structure of SARS-CoV-2 main protease provides a basis for design of improved α-ketoamide inhibitors

**DOI:** 10.1126/science.abb3405

**Published:** 2020-03-20

**Authors:** Linlin Zhang, Daizong Lin, Xinyuanyuan Sun, Ute Curth, Christian Drosten, Lucie Sauerhering, Stephan Becker, Katharina Rox, Rolf Hilgenfeld

**Affiliations:** 1 Institute of Biochemistry, Center for Structural and Cell Biology in Medicine, University of Lübeck, 23562 Lübeck, Germany.; 2 German Center for Infection Research (DZIF), Hamburg-Lübeck-Borstel-Riems Site, University of Lübeck, 23562 Lübeck, Germany.; 3 Changchun Discovery Sciences Ltd., 789 Shunda Road, Changchun, Jilin 130012, China.; 4 Institute for Biophysical Chemistry, Hannover Medical School, 30625 Hannover, Germany.; 5 Institute of Virology, Charité Universitätsmedizin Berlin, 10117 Berlin, Germany.; 6 Institute of Virology, University of Marburg, 35043 Marburg, Germany.; 7 German Center for Infection Research (DZIF), Marburg-Gießen-Langen Site, University of Marburg, 35043 Marburg, Germany.; 8 Department of Chemical Biology, Helmholtz Center for Infection Research (HZI), 38124 Braunschweig, Germany.; 9 German Center for Infection Research (DZIF), Hannover-Braunschweig Site, Helmholtz Center for Infection Research, 38124 Braunschweig, Germany.

## Abstract

The coronavirus disease 2019 (COVID-19) pandemic caused by severe acute respiratory syndrome–coronavirus 2 (SARS-CoV-2) is a global health emergency. An attractive drug target among coronaviruses is the main protease (M^pro^, also called 3CL^pro^) because of its essential role in processing the polyproteins that are translated from the viral RNA. We report the x-ray structures of the unliganded SARS-CoV-2 M^pro^ and its complex with an α-ketoamide inhibitor. This was derived from a previously designed inhibitor but with the P3-P2 amide bond incorporated into a pyridone ring to enhance the half-life of the compound in plasma. On the basis of the unliganded structure, we developed the lead compound into a potent inhibitor of the SARS-CoV-2 M^pro^. The pharmacokinetic characterization of the optimized inhibitor reveals a pronounced lung tropism and suitability for administration by the inhalative route.

## Targeting a key enzyme in SARS-CoV-2

Scientists across the world are working to understand severe acute respiratory syndrome–coronavirus 2 (SARS-CoV-2), the virus that causes coronavirus disease 2019 (COVID-19). Zhang *et al.* determined the x-ray crystal structure of a key protein in the virus' life cycle: the main protease. This enzyme cuts the polyproteins translated from viral RNA to yield functional viral proteins. The authors also developed a lead compound into a potent inhibitor and obtained a structure with the inhibitor bound, work that may provide a basis for development of anticoronaviral drugs.

## Teaser

Optimized inhibitor of a key enzyme of the novel coronavirus exhibits pronounced lung tropism.

In December 2019, a new coronavirus caused an outbreak of pulmonary disease in the city of Wuhan, the capital of Hubei province in China, and has since spread globally (*1*, *2*). The virus has been named severe acute respiratory syndrome–coronavirus 2 (SARS-CoV-2) (*3*) because the RNA genome is about 82% identical to that of the SARS coronavirus (SARS-CoV); both viruses belong to clade b of the genus *Betacoronavirus *(*1*, *2*). The disease caused by SARS-CoV-2 is called coronavirus disease 2019 (COVID-19). Whereas at the beginning of the outbreak, cases were connected to the Huanan seafood and animal market in Wuhan, efficient human-to-human transmission led to exponential growth in the number of cases. On 11 March 2020, the World Health Organization (WHO) declared the outbreak a pandemic. As of 9 April, there were >1,500,000 cumulative cases globally, with a ~5.9% case fatality rate.

One of the best-characterized drug targets among coronaviruses is the main protease (M^pro^, also called 3CL^pro^) (*4*). Along with the papain-like protease(s), this enzyme is essential for processing the polyproteins that are translated from the viral RNA (*5*)*.* The M^pro^ operates at no fewer than 11 cleavage sites on the large polyprotein 1ab (replicase 1ab, ~790 kDa); the recognition sequence at most sites is Leu-Gln↓(Ser, Ala, Gly) (↓ marks the cleavage site). Inhibiting the activity of this enzyme would block viral replication. Because no human proteases with a similar cleavage specificity are known, such inhibitors are unlikely to be toxic.

**Fig. 1:**
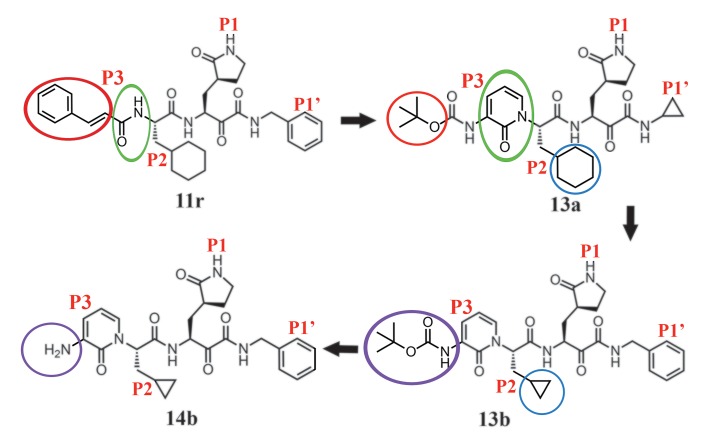
Chemical structures of α-ketoamide inhibitors 11r, 13a, 13b, and 14b. Colored ovals and circles highlight the modifications from one development step to the next (see text).

Previously, we designed and synthesized peptidomimetic α-ketoamides as broad-spectrum inhibitors of the main proteases of betacoronaviruses and alphacoronaviruses as well as the 3C proteases of enteroviruses (*6*). The best of these compounds (**11r**; Fig. 1) showed an half-maximal effective concentration (EC_50_) of 400 pM against Middle East respiratory syndrome–coronavirus (MERS-CoV) in Huh7 cells as well as low-μM EC_50_ values against SARS-CoV and a whole range of enteroviruses in various cell lines, although the antiviral activity seemed to depend to a great extent on the cell type used in the experiments (*6*). To improve the half-life of the compound in plasma, we modified **11r** by hiding the P3-P2 amide bond within a pyridone ring (Fig. 1, green ovals) in the expectation that this might prevent cellular proteases from accessing this bond and cleaving it. Further, to increase the solubility of the compound in plasma and to reduce its binding to plasma proteins, we replaced the hydrophobic cinnamoyl moiety by the somewhat less hydrophobic Boc group (Fig. 1, red ovals) to give **13a** (see scheme S1 for synthesis).

**Fig. 2:**
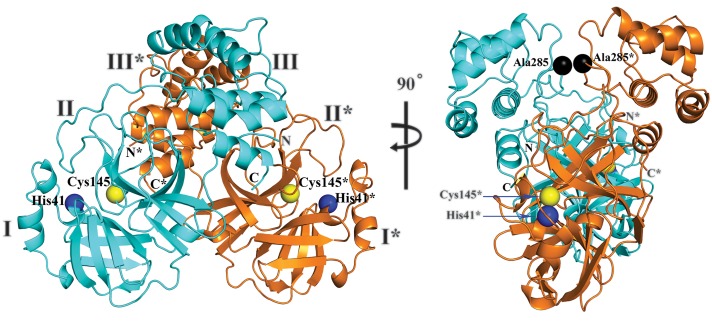
Three-dimensional structure of SARS-CoV-2 M^pro^ in two different views. One protomer of the dimer is shown in light blue, the other one in orange. Domains are labeled by Roman numerals. Amino acid residues of the catalytic site are indicated as yellow spheres for Cys^145^ and blue spheres for His^41^. Asterisks mark residues from protomer B (orange). Black spheres indicate the positions of Ala^285^ for each of the two domains III (see text). Chain termini are labeled N and C for molecule A (light blue) and N* and C* for molecule B (orange).

To examine whether the introduced pyridone ring is compatible with the three-dimensional structure of the target, we determined the crystal structure, at 1.75 Å resolution, of the M^pro^ of SARS-CoV-2 (Fig. 2). The three-dimensional structure is highly similar to that of the SARS-CoV M^pro^, as expected from the 96% sequence identity (see fig. S8); the root mean square deviation between the two free-enzyme structures is 0.53 Å for all Cα positions [comparison between SARS-CoV-2 M^pro^ structure and SARS-CoV M^pro^, PDB entry 2BX4 (*7*)]. The chymotrypsin-like and picornavirus 3C protease–like domains I and II (residues 10 to 99 and 100 to 182, respectively) are six-stranded antiparallel β barrels that harbor the substrate-binding site between them. Domain III (residues 198 to 303), a globular cluster of five helices, is involved in regulating the dimerization of the M^pro^, mainly through a salt-bridge interaction between Glu^290^ of one protomer and Arg^4^ of the other (*8*). The tight dimer formed by SARS-CoV-2 M^pro^ has a contact interface of ~1394 Å^2^, predominantly between domain II of molecule A and the NH_2_-terminal residues (“N-finger”) of molecule B, with the two molecules oriented perpendicular to one another (Fig. 2). Dimerization of the enzyme is necessary for catalytic activity, because the N-finger of each of the two protomers interacts with Glu^166^ of the other protomer and thereby helps shape the S1 pocket of the substrate-binding site (*9*). To reach this interaction site, the N-finger is squeezed in between domains II and III of the parent monomer and domain II of the other monomer.

Interestingly, in the SARS-CoV but not in the SARS-CoV-2 M^pro^ dimer, there is a polar interaction between the two domains III involving a 2.60-Å hydrogen bond between the side-chain hydroxyl groups of residue Thr^285^ of each protomer, supported by a hydrophobic contact between the side chain of Ile^286^ and Thr^285^ Cγ2. In SARS-CoV-2, the threonine is replaced by alanine (indicated by the black spheres in Fig. 2) and the isoleucine by leucine (fig. S8). It was previously shown that replacing Ser^284^, Thr^285^, and Ile^286^ by alanine residues in SARS-CoV M^pro^ leads to enhancement of the catalytic activity of the protease by a factor of 3.6, concomitant with a slightly closer packing of the two domains III of the dimer against one another (*10*). This was accompanied by changes in enzyme dynamics that transmit the effect of the mutation to the catalytic center. Indeed, the Thr^285^ → Ala replacement observed in the SARS-CoV-2 M^pro^ also allows the two domains III to approach each other more closely (the distance between the Cα atoms of residues 285 in molecules A and B is 6.77 Å in SARS-CoV M^pro^ and 5.21 Å in SARS-CoV-2 M^pro^, and the distance between the centers of mass of the two domains III shrinks from 33.4 Å to 32.1 Å). However, the catalytic efficiency of SARS-CoV-2 M^pro^ is only slightly higher, if at all [turnover number (*k*_cat_)/Michaelis constant (*K*_m_) = 3426.1 ± 416.9 s^–1^ M^–1^] than that of SARS-CoV M^pro^ (*k*_cat_/*K*_m_ = 3011.3 ± 294.6 s^–1^ M^–1^). Further, the estimated dissociation constant of dimerization is the same (~2.5 μM) for the two enzymes, as determined by analytical ultracentrifugation (fig. S10).

**Fig. 3:**
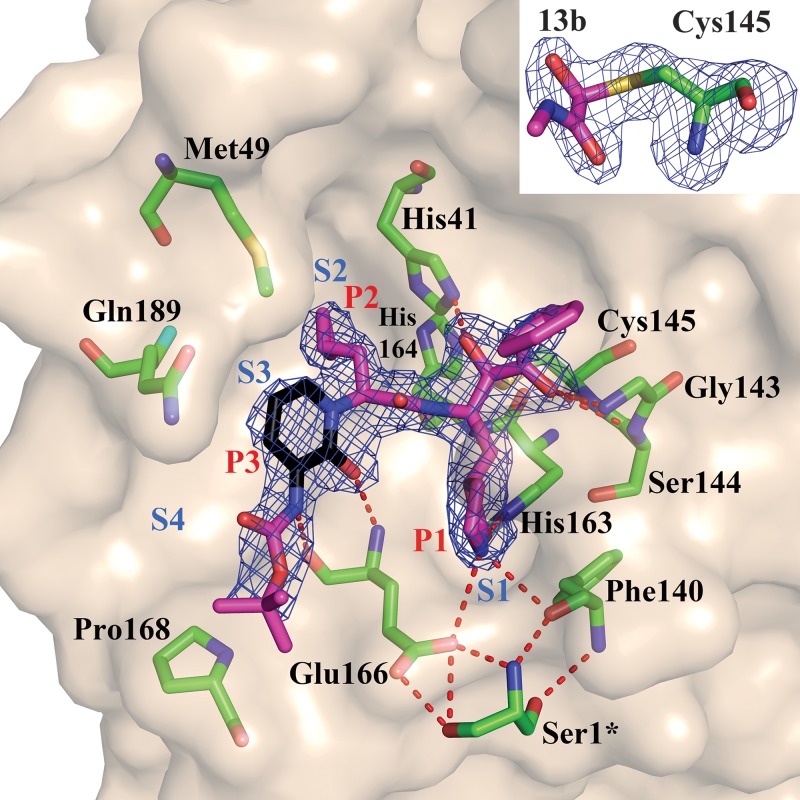
Compound 13b in the substrate-binding cleft located between domains I and II of the M^pro^ in the monoclinic crystal form (space group *C*2). *F*_obs_ – *F*_calc_ density is shown for the inhibitor (contouring level 3σ). Carbon atoms of the inhibitor are magenta, except in the pyridone ring, which is black; oxygen atoms are red, nitrogens blue, and sulfur yellow. Light blue symbols S*n* (*n* = 1, 2, 3…) indicate the canonical binding pockets for moieties P*n* (*n* = 1, 2, 3…) (red symbols) of the peptidomimetic inhibitor. Hydrogen bonds are indicated by dashed red lines. Note the interaction between Ser^1^*, the N-terminal residue of molecule B, and Glu^166^ of molecule A, which is essential for keeping the S1 pocket in the correct shape and the enzyme in the active conformation. Inset: Thiohemiketal formed by the nucleophilic attack of the catalytic cysteine onto the α-carbon of the inhibitor. The stereochemistry of the α-carbon is *S*. *F*_obs_ − *F*_calc_ density (contoured at 3σ) is shown in blue. See fig. S9 for more details.

We used this crystal structure to dock the α-ketoamide **13a**; this suggested that the pyridone ring might have some steric clash with the side chain of Gln^189^. However, in our previous work (*6*), we had found Gln^189^ to be quite flexible, and therefore we went ahead with **13a** as a lead. The plasma half-life of this compound in mice was increased by a factor of ~3 relative to **11r** (from 0.3 hours to 1.0 hours), the in vitro kinetic plasma solubility was improved by a factor of ~19 (from 6 μM for **11r** to 112 μM for **13a**), and the thermodynamic solubility increased by a factor of ~13 (from 41 μM to 530 μM). Binding to mouse plasma protein was reduced from 99% to 97% [many drugs have plasma protein binding of >90% (*11*)]. However, relative to **11r** (IC_50_ = 0.18 ± 0.02 μM), the structural modification led to some loss of inhibitory activity against the main protease of SARS-CoV-2 (IC_50_ = 2.39 ± 0.63 μM) as well as the 3C proteases (3C^pro^) of enteroviruses. **11r** was designed for broad-spectrum activity, with the P2 cyclohexyl moiety intended to fill a pocket in the enterovirus 3C^pro^. The S2 pocket of the betacoronavirus M^pro^ (Fig. 3) features substantial plasticity, enabling it to adapt to the shape of smaller inhibitor moieties (*6*). To enhance the antiviral activity against betacoronaviruses of clade b (SARS-CoV-2 and SARS-CoV), we sacrificed the goal of broad-spectrum activity and replaced the P2 cyclohexyl moiety of **13a** by the smaller cyclopropyl in **13b** (Fig. 1, blue circles). Here, we present x-ray crystal structures in two different crystal forms, at 1.95 and 2.20 Å resolution, of the complex between α-ketoamide **13b** and the M^pro^ of SARS-CoV-2. One structure is in space group *C*2 (Fig. 3), where both protomers of the M^pro^ dimer are bound by crystal symmetry to have identical conformations; the other is in space group *P*2_1_2_1_2_1_, where the two protomers are independent of each other and free to adopt different conformations. Indeed, we find that in the latter crystal structure, the key residue Glu^166^ adopts an inactive conformation in protomer B (as evidenced by its distance from His^172^ and the lack of H-bonding interaction with the P1 moiety of the inhibitor), even though compound **13b** is bound in the same mode as in molecule A. This phenomenon has also been observed with the SARS-CoV M^pro^ (*12*) and is consistent with the half-site activity described for this enzyme (*13*). In all copies of the inhibited SARS-CoV-2 M^pro^, the inhibitor binds to the shallow substrate-binding site at the surface of each protomer, between domains I and II (Fig. 3).

Through the nucleophilic attack of the catalytic Cys^145^ onto the α-keto group of the inhibitor, a thiohemiketal is formed in a reversible reaction. This is clearly reflected in the electron density (Fig. 3, inset); the stereochemistry of this chiral moiety is *S* in all copies of compound **13b** in these structures. The oxyanion (or hydroxyl) group of this thiohemiketal is stabilized by a hydrogen bond from His^41^, whereas the amide oxygen of **13b** accepts a hydrogen bond from the main-chain amides of Gly^143^, Cys^145^, and partly Ser^144^, which form the canonical “oxyanion hole” of the cysteine protease. It is an advantage of the α-ketoamides that their warhead can interact with the catalytic center of the target proteases through two hydrogen-bonding interactions (*6*) rather than only one, as with other warheads such as aldehydes (*14*) or Michael acceptors (*15*)*.*

The P1 γ-lactam moiety, designed as a glutamine surrogate (*15*, *16*), is deeply embedded in the S1 pocket of the protease, where the lactam nitrogen donates a three-center (bifurcated) hydrogen bond to the main-chain oxygen of Phe^140^ (3.20/3.10/3.28 Å; values for the structure in space group *C*2/space group *P*2_1_2_1_2_1_ molecule A/space group *P*2_1_2_1_2_1_ molecule B) and to the Glu^166^ carboxylate [3.35/3.33/(3.55) Å], and the carbonyl oxygen accepts a 2.57/2.51/2.81 Å hydrogen bond from the imidazole of His^163^. The P2 cyclopropyl methyl moiety fits snugly into the S2 subsite, which has shrunk by 28 Å^3^ relative to the complex between compound **13a** with P2 = cyclohexyl methyl and the SARS-CoV M^pro^ (*17*). The pyridone in the P3-P2 position of the inhibitor occupies the space normally filled by the substrate’s main chain; its carbonyl oxygen accepts a 2.89/2.99/3.00 Å hydrogen bond from the main-chain amide of residue Glu^166^. Further, the P3 amide donates a 2.83/2.96/2.87 Å hydrogen bond to the main-chain oxygen of Glu^166^. Embedded within the pyridone, the P2 nitrogen can no longer donate a hydrogen bond to the protein (the H-bond prevented from forming would connect the P2 nitrogen and the side-chain oxygen of Gln^189^; these two atoms are highlighted in fig. S9). However, our previous crystal structures showed that the P2 main-chain amide of the linear α-ketoamides does not make a hydrogen bond with the protein in all cases, so this interaction does not seem to be critical (*6*). The protecting Boc group on P3 does not occupy the canonical S4 site of the protease [in contrast to the protecting groups of other inhibitors in complex with the SARS-CoV M^pro^ (*18*)] but is located near Pro^168^ (3.81/4.17/3.65 Å) (Fig. 3); as a result of this interaction, the latter residue moves outward by more than 2 Å (relative to the structure of the free enzyme). This contact explains why removing the Boc group as in compound **14b** (Fig. 1, purple ovals) weakens the inhibitory potency of this compound by a factor of ~2. Interestingly, there is a space between the pyridone ring of **13b**, the main chain of residue Thr^190^, and the side chain of Gln^189^ (smallest distance: 3.6 Å), which is filled by a dimethyl sulfoxide (DMSO) molecule in the *C*2 crystal structure and a water molecule in the *P*2_1_2_1_2_1_ structure. This suggests that P3 moieties more bulky than pyridone may be accepted here.

**Fig. 4:**
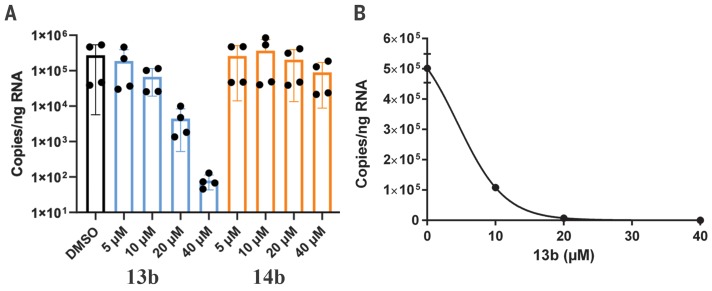
Compound 13b inhibits SARS-CoV-2 replication in human Calu-3 lung cells. (**A**) Calu-3 cells were infected with SARS-CoV-2 using a multiplicity of infection (MOI) of 0.05. Varying amounts (5, 10, 20, or 40 μM) of **13b** (blue bars) or **14b** (orange bars) were added. DMSO was used as vehicle control (black bar). Total RNA was isolated from cell lysates, and viral RNA content was analyzed by quantitative polymerase chain reaction. Data are means ± SD of two biological experiments with two technical replicates each. (**B**) For the estimation of the EC_50_ value of compound **13b** against SARS-CoV-2, a dose-response curve was prepared (GraphPad).

Compound **13b** inhibits the purified recombinant SARS-CoV-2 M^pro^ with IC_50_ = 0.67 ± 0.18 μM. The corresponding IC_50_ values for inhibition of the SARS-CoV M^pro^ and the MERS-CoV M^pro^ are 0.90 ± 0.29 μM and 0.58 ± 0.22 μM, respectively. In a SARS-CoV replicon (*19*), RNA replication is inhibited with EC_50_ = 1.75 ± 0.25 μM. In human Calu-3 cells infected with SARS-CoV-2, an EC_50_ of 4 to 5 μM was observed, whereas compound **14b** lacking the Boc group was almost inactive (Fig. 4). This suggests that the hydrophobic and bulky Boc group is necessary to cross the cellular membrane and that an even more hydrophobic moiety might be advantageous here, although this may again lead to increased plasma protein binding, as observed for the cinnamoyl-containing **11r**.

To assess the absorption-distribution-metabolism-excretion (ADME) properties of the pyridone-containing α-ketoamides, we first investigated compound **13a**. Metabolic stability in mouse and human microsomes was good, with intrinsic clearance rates Cl_int_mouse_ = 32.0 μl min^–1^ (mg protein)^–1^ and Cl_int_human_ = 21.0 μl min^–1^ (mg protein)^–1^. This means that after 30 min, ~80% and 60% (for mouse and human, respectively) of residual compound remained metabolically stable. Pharmacokinetic studies in CD-1 mice using the subcutaneous route at 20 mg/kg showed that **13a** stayed in plasma for up to 4 hours but was excreted via urine for up to 24 hours. The maximum plasma concentration (*C*_max_) was determined at 334.5 ng ml^−1^ and the mean residence time was ~1.6 hours. Although **13a** seemed to be cleared very rapidly from plasma, at 24 hours it was found at 135 ng/g tissue in the lung and at 52.7 ng ml^−1^ in bronchio-alveolar lavage fluid (BALF), which suggests that it was mainly distributed to tissue. Next, we investigated **13b** for its pharmacokinetic properties in CD-1 mice using the subcutaneous route as well, but at 3 mg kg^−1^. The ADME parameters of **13b** were similar to those of **13a**; in addition, binding to human plasma proteins was found to be 90%. The *C*_max_ of **13b** was determined at 126.2 ng ml^−1^. This is around 37% of the *C*_max_ detected for **13a**, although the **13b** dosage was lower by a factor of ~7. The mean residence time for **13b** was extended to 2.7 hours and the plasma half-life in mice was 1.8 hours. In addition, **13b** showed a less rapid clearance relative to **13a** (table S3). During the pharmacokinetic study with **13b**, we monitored its lung tissue levels. After 4 hours, **13b** was still found at ~13 ng g^−1^ in lung tissue. This lung tropism of **13a** and **13b** is beneficial given that COVID-19 affects the lungs. In addition to subcutaneous administration, **13b** was nebulized using an inhalation device at 3 mg kg^−1^. After 24 hours, **13b** was found at 33 ng g^−1^ in lung tissue. Inhalation was tolerated well and mice did not show any adverse effects, which suggests that direct administration of the compound to the lungs would be possible. Given these favorable pharmacokinetic results, our study provides a useful framework for the development of the pyridone-containing inhibitors toward anticoronaviral drugs.

## Supplementary Materials

science.sciencemag.org/content/368/6489/409/suppl/DC1 Materials and Methods Supplementary Text Scheme S1 Figs. S1 to S10 Tables S1 to S3 References (*20*–*42*)

## Appendix

View/request a protocol for this paper from *Bio-protocol*.
